# Degradation Characteristics of Cellulose Acetate in Different Aqueous Conditions

**DOI:** 10.3390/polym15234505

**Published:** 2023-11-23

**Authors:** Jiao Tan, Yinchun Liang, Lihui Sun, Zhanping Yang, Jingjing Xu, Dejun Dong, Huan Liu

**Affiliations:** 1 State Key Laboratory of Food Science and Resources, China-Canada Joint Laboratory of Food Science and Technology (Nanchang), Key Laboratory of Bioactive Polysaccharides of Jiangxi Province, Nanchang University, Nanchang 330047, China; tanjiao202309@163.com; 2 Nantong Cellulose·Fibers Company Co., Ltd., Nantong 226008, China

**Keywords:** cellulose acetate, different aqueous conditions, degradation

## Abstract

Cellulose acetate (CA) is widely used in cigarette filters and packaging films, but due to its acetylation, it is difficult to degrade in the natural environment, and the problem of pollution has become a serious challenge. Understanding the degradation behavior and performance of CA in different environments is the basis and prerequisite for achieving its comprehensive utilization and developing efficient degradation methods. In this study, we investigated the degradation performance of CA in different aqueous environments to evaluate the effects of pH, salinity and microorganisms on CA degradation. The CA tows and films were immersed in HCl, NaOH solution, river water, seawater or homemade seawater for 16 weeks and the degradation mechanism was investigated by the changes in weight loss rate, degree of substitution, hydrophilicity, molecular structure and surface morphology. The results showed that the degradation of CA tow and film were the fastest in NaOH solution; the weight loss rates after 16 weeks were 40.29% and 39.63%, respectively, followed by HCl solution, and the degradation performance of CA tow was better than that of film. After 16 weeks of degradation in river water, seawater and homemade seawater, all the weight loss rates were less than 3%. In summary, this study illustrated that the environmental acidity, basicity and high concentration of inorganic salts had a critical promotion effect on the non-enzymatic hydrolysis of CA, whereas the number and type of microorganisms were the key factors affecting the biodegradation of CA.

## 1. Introduction

Cellulose acetate (CA) is mainly made from cellulose material (high content of α-cellulose) obtained from wood-dissolved pulp or cotton [1]. CA is the first commercially produced and continuously developing cellulose organic ester among cellulose derivatives [2]. According to the degree of substitution, cellulose acetate can mainly be divided into three categories, cellulose acetate (CMA), cellulose diacetate (CDA), and cellulose triacetate (CTA) [3]. CA has good biocompatibility, chemical and thermal stability, with a low price, excellent performance, and a wide range of applications [4]. At present, CA is mainly used in cigarette filters [5,6], membrane materials [7,8], packaging material [9,10] and textiles [11]. However, due to the acetylation of cellulose, CA is difficult to degrade in the natural environment and can cause serious environmental impacts. For instance, Joly et al. [12] conducted a composting experiment on CA-based cigarette holder waste for six months and found that the mass loss rate of cigarette holders was only 20% after six months.

At present, the degradation methods of CA in the natural environment mainly include hydrolytic degradation, photodegradation and biodegradation. Under the action of hydrolytic degradation, the main acting site is the ester bonds and β-1,4 glucoside bonds in the CA matrix. The destruction of ester bonds will reduce the degree of substitution and at the same time produce acetic acid, which may accelerate the degradation process of CA [13]. Ultraviolet radiation can cause photooxidative degradation, generating free radicals that decompose CA into small fragments with reduced molecular weight [14]. For microbial degradation, the microorganisms capable of degrading CA first adhere to the surface of the matrix and then secrete specific enzymes, and finally biodegrade CA into small molecular substances or their fragments [15]. The degradation rate of CA is highly related to its structure, such as degree of substitution, degree of crystallinity and molecular weight [16]. Jadav et al. [17] studied the UV-A radiation degradation process of carbon point (CDs) modified cellulose acetate (CA + CD) films in air and simulated seawater, and the results showed that the addition of CDs accelerated the deacetylation of CA, after being irradiated in air and simulated seawater for 30 days, the weightlessness of CA + CD was 53% and 43%, respectively, while that of ordinary CA films was only 12% and 4%, respectively. Park et al. [18] studied the degradation performance of linen fabric, acetate, cotton and rayon by the soil-buried degradation method and found that linen fabric had the strongest biodegradation performance, while acetate still maintained its original shape.

Due to its stability and durability, CA may persist in the environment for several or even dozens of years. Studying the degradation performance of CA in different aqueous environments is conducive to the in-depth understanding of the degradation rules of CA, which will provide a certain theoretical basis for the establishment of CA rapid degradation performance evaluation methods and has important guiding significance for solving the problem of CA environmental pollution. Among the existing literature, major studies on the degradation performance of CA in composting and photodegradation were performed, however, few studies were conducted on the degradation performance of CA in different aqueous conditions. Additionally, the degradation effect of different forms of CA in different aqueous conditions is unclear, which represents a certain obstacle to the development of rapid degradation methods and the expansion of their application range. Thus, in this research, we mainly focused on the degradation behavior of CA in different aqueous conditions (HCl solution, NaOH solution, river water, seawater and homemade seawater) and analyzed the weight loss rate, substitution degree, hydrophilicity, morphology and chemical structure variations of the corresponding CA degradation residues during degradation.

## 2. Materials and Methods

### 2.1. Materials

Cellulose acetate (CA, DS = 2.45, DP = 317) tow and vinegar tablets were obtained from Nantong Acetate Fiber Co., Ltd. (Nantong, China). Acetone was obtained from Sinopharm Chemical Reagent Co., Ltd. (Shanghai, China). Hydrochloric acid standard solution (HCl, 0.5 mol/L), standard solution of sodium hydroxide (NaOH, 0.5 mol/L), Phenolphthalein indicator, sodium chloride (NaCl), magnesium chloride (MgCl_2_), potassium bromide (KBr) and other reagents were purchased from Aladdin Biological Technology Co., Ltd. (Shanghai, China).

### 2.2. Preparation of CA Film

Solvent evaporation was utilized for the preparation of CA film. CA was dissolved in acetone at a ratio of 1:10 (*w*/*v*) and stirred with a magnetic stirrer until completely dissolved, then the dispersion was sonicated in an ultrasonic bath for half an hour and left overnight. Next, an equal amount of defoaming casting solution was poured into a petri dish (9 cm in diameter), ensuring even distribution and using a scraper to achieve the desired thickness. The scraped film was evaporated in a fume hood for 1 h and subsequently air-dried at room temperature to make a CA film. The film was opened with a needle and gently peeled off.

### 2.3. Degradation Behavior of CA in Different Aqueous Conditions

#### 2.3.1. Room Temperature Hydrolysis

The CA tow and film with constant weight were taken and hydrolyzed in HCl solution (pH = 1) and NaOH solution (pH = 12) at room temperature. The pH value of NaOH solution was monitored in real time and the solution was replaced in time. The experimental period was 16 weeks, and the samples were taken out at certain intervals, washed repeatedly with distilled water and dried to a constant weight.

#### 2.3.2. Degradation in Different Aqueous Environments

The CA tow and film with constant weight were taken and placed in static river water, seawater and homemade seawater, respectively. Different water sources were placed in glassware, and the water surface height was recorded with the scale line. During the experiment, due to the continuous evaporation of the water body, the method of adding distilled water was used to maintain the water’s surface height. The water samples were changed every 15 days. The experimental period was 16 weeks, and the samples were taken out at certain intervals, washed repeatedly with distilled water and dried to a constant weight. The chemical composition of homemade seawater is shown in Table 1.

### 2.4. Weight Loss Determination

The CA tow and film were degraded in different aqueous environments for 16 weeks, and CA was taken out at intervals, washed with distilled water and dried to constant weight. The percent weight loss was calculated as follows:

(1) $$\mathrm{Weight}\;\mathrm{loss}\;(\%)=\frac{m_{0}-m}{m_{0}}\times 100\%$$

where m_0_ was the initial dry weight of CA, m was the dry weight after a given time of degradation.

### 2.5. DS Determination

The degree of substitution (DS) of CA tow and film before and after degradation was determined by referring to ASTM D 871-96 [20]. The sample of 0.1 g CA was accurately weighed and dried at 105 ℃ to constant weight in an air blast drying oven. Then the sample was put into a 100 mL conical flask with a piston, adding 20 mL acetone, adding 5 mL 0.5 mol/L NaOH standard solution, and reacted at room temperature for 2 h. The excess NaOH standard solution was titrated with 0.5 mol/L HCl standard solution, using phenolphthalein as the indicator. Add 1 mL of 0.5 mol/L HCl standard solution and allow the NaOH standard solution to diffuse from CA for a period of time. Next, a small amount of excess HCl was titrated with a standard solution of 0.5 mol/L NaOH standard solution. A blank test was performed in the same manner except that the CA sample was not added. Calculate the percentage content of combined acetyl as follows:

(2) $$W\%=\frac{\left[\left(D-C\right)N_{a}+\left(A-B\right)N_{b}\right]\times 4.305}{w}$$

where:

A—NaOH standard solution titrates the volume of the sample, mL,
B—NaOH standard solution titrates the volume of the blank, mL,
N_b_—normality of the NaOH standard solution, mol/L,
C—HCl standard solution titrates the volume of the sample, mL,
D—HCl standard solution titrates the volume of the blank, mL,
N_a_—normality of the HCl standard solution, mol/L, and
w—CA sample used, g.

(3) $$\mathrm{DS}=\frac{3.86\;W}{102.4-W}$$

where DS was the degree of substitution of the sample.

### 2.6. Determination of Water Retention Value (WRV) and Water Absorption (WAV)

The dry CA tow of 0.1 g was accurately weighed and placed in a centrifuge tube containing 30 mL of distilled water, making the sample completely immersed in a sufficient amount of distilled water for 24 h. The damp tow was centrifuged at 3000× *g* for 30 min to remove excess water and weighed. The samples were then dried to a constant weight at 105 °C in a blast dryer, cooled to room temperature in a dryer and reweighed. The water retention value of the samples was calculated using the following formula:

(4) $$\mathrm{WRV}=\frac{W_{1}-W_{2}}{W_{2}}\times 100\%$$

where W_1_ was the weight of the wet sample after centrifugation, and W_2_ was the weight of the dry sample after drying.

The dry CA film of 0.1 g was weighed to constant weight at 80 °C in a blast dryer, cooled to room temperature in a dryer and weighed. The film was placed in a conical bottle containing 50 mL distilled water, making CA film completely immersed in a sufficient amount of distilled water for 24 h. Then dry the water on the surface of CA film and weigh the wet sample. The formula for calculating water absorption was as follows:

(5) $$\mathrm{WAV}=\frac{W_{2}-W_{1}}{W_{1}}\times 100\%$$

where W_1_ was the weight of the dry sample, and W_2_ was the weight of the wet sample.

### 2.7. Fourier-Transform Infrared Spectroscopy (FT-IR)

The molecular structure of the CA before and after degradation was obtained by Fourier-transform infrared spectroscopy (Nicolet 5700, Thermo Fisher Scientific, Waltham, MA, USA). The CA samples were thoroughly mixed and ground with KBr at a mass ratio of 1:100, and then pressed into powder. A total of 32 scans were recorded in the wavenumber area of 400 to 4000 cm^−1^.

### 2.8. Scanning Electron Microscopy (SEM)

The morphology of CA samples before and after degradation was observed by scanning electron microscope (SU8010, Hitachi, Tokyo, Japan) under 3 kV electron beam acceleration voltage. Before observation, an appropriate amount of CA tow and film were smeared on the conductive silica gel, and the CA surface was treated with gold spraying instrument.

### 2.9. Statistical Analysis

All measurements were carried out in triplicate. Data were processed and analyzed by SPSS Statistics Software 24.0. Statistical analysis was performed by one-way ANOVA. The differences were considered significant when *p* < 0.05. Origin 2021 was used to analyze data and form graphs.

## 3. Results and Discussion

### 3.1. Weight Loss Rates of CA in Different Aqueous Conditions

**Figure 1:**
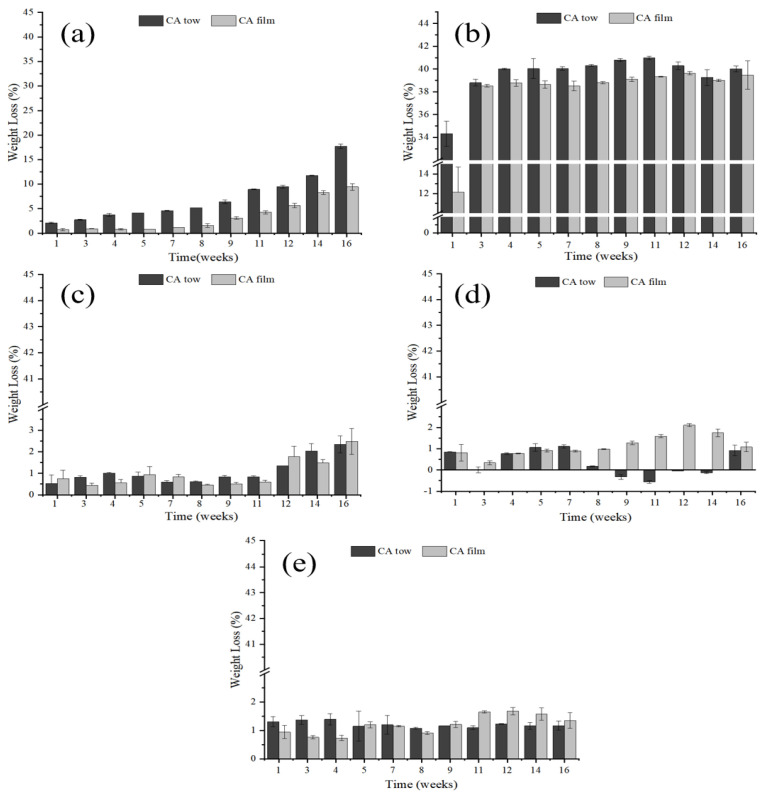
Weight loss rates of CA in different conditions ((**a**): HCl solution; (**b**): NaOH solution; (**c**): river water; (**d**): seawater; (**e**): homemade seawater).

The weight loss rate of CA tow and film in different aqueous conditions are shown in Figure 1. In the HCl environment, with the increase in degradation time, the weight loss rates of both the tow and the film increased slowly, as H^+^ can promote the reaction of ester bond hydrolysis in CA, in which the weight losses of CA tow increased from 2.07% to 17.71% and the CA film increased from 0.72% to 9.44%. At the same degradation time, the weight loss rate of CA tow was significantly higher than that of the CA film, which could be attributed to the fact that the contact area between CA tow and water phase was larger than that of the CA film leading to a faster hydrolysis rate. When CA was degraded in NaOH solution, the weight loss rate of the tow and film increased rapidly within 1–3 weeks, however, there was no significant change in weight loss rate after 3 weeks, which could be attributed to the fact that OH^-^ would attack the carbon-based carbon atoms in CA and promote the breaking of ester bonds in CA, thus accelerating the degradation [21]. After the third week, the acetyl groups in CA had been basically removed, and the OH^-^ mainly acted on the ester bond without destroying the glycosidic bond, so the weight loss rate remained unchanged. A similar phenomenon was also observed by Yamashita et al., who found that when CA film with high substitution degree were degraded in low concentration acid-base environment, the sample weight loss decreased faster in alkaline environment than in acidic environment [22]. Compared with HCl and NaOH environments, the weight losses of CA tow and film in river water, seawater and homemade seawater conditions were significantly lower. When CA was degraded in river water, the weight loss rates of both tow and film increased slowly with the increase of degradation time. As shown in Figure 1c, only 2.34% and 2.48% mass losses of CA tow and film in river water conditions were observed after 16 weeks, respectively, which may be due to the gradual adhesion of microorganisms on the surface of CA and initiating of secretion of specific enzymes to produce degradation sites, thereby removing part of the acetyl group [23]. During the degradation of CA in seawater and homemade seawater, the weight loss rates of the CA tow and film were rarely altered with the increase in degradation time. Similar result could also be found in the study of Tsuji et al., who studied the degradation of PLLA in static seawater and found that no obvious weight loss was observed in PLLA film after 10 weeks of degradation [24].

### 3.2. DS variations of CA in Different Aqueous Conditions

The degree of substitution of CA tow and film degraded in different aqueous environments were shown in Table 2. The degree of substitution refers to the average number of active hydroxyl groups replaced on each glucose unit of cellulose. Since only three hydroxyl groups can be replaced in each dehydrated glucose unit in the cellulose molecular chain, the degree of substitution can only be less than or equal to 3 [25]. The higher the degree of substitution, the worse the biodegradability of CA [26]. In HCl solution, after degradation of 16 weeks, the degree of substitution of tow and film decreased from 2.45 to 1.33 and 1.78, respectively. During the degradation of CA in NaOH solution, the degree of substitution decreased the fastest, and the acetyl groups of tow and film were removed in 1 week and 3 weeks, respectively. A similar phenomenon was also observed by Sailema-Palate et al., who found that PCL films degraded faster in NaOH solution than in HCl solution [27]. When CA was degraded in river water, seawater and homemade seawater, the degree of substitution slightly decreased with the increase in degradation time. However, the acetyl group removal rate of CA in river water was faster than that in seawater and homemade seawater, which may be due to the fact that the number and species of microorganisms in seawater and homemade seawater were less than in river water, and the secretion rate of enzymes was very slow, resulting in a slow degradation process. It had been reported that the number and species composition of microorganisms in seawater varies by location and depth, with bacterial density generally ranging from 10^4^ to 10^6^ per milliliter on average [28,29].

### 3.3. Hydrophilic Properties of CA Degraded by Different Aqueous Conditions

**Figure 2:**
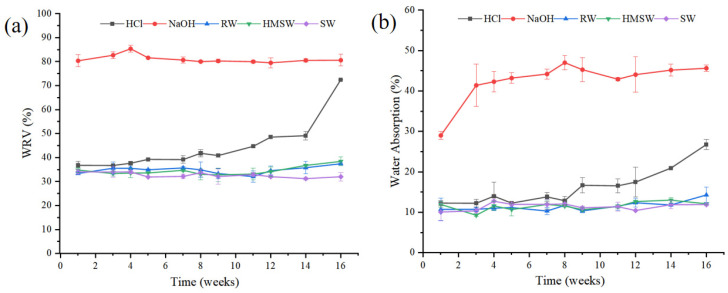
Water retention values and water absorption rates of CA tow (**a**) and film (**b**) in different conditions.

WRV can characterize the wetting degree of cellulose and indirectly characterize the accessibility of cellulose to enzymes [30]. WRV is the amount of water retained by the substrate after centrifugation, so this method can be used to estimate the total amount of water interacting with a given substrate and as a predictor of hydrolysis yield [31,32]. The WRV of the CA tow degraded in different aqueous environments were shown in Figure 2a. When CA was degraded in the HCl environment, the WRV increased gradually with the increase in degradation time (from 34.02% to 72.48%). When CA was degraded in NaOH environment for one week, the WRV rapidly reached 80.35%, and no significant change was found afterward, which could be due to the fact that the acetyl groups had been completely removed. When CA was degraded in river water, seawater and homemade seawater, the WRV did not change significantly with the increase in degradation time.

The water absorption rates of CA film degraded in different aqueous environments are shown in Figure 2b. Studies have shown that the increase in water absorption is conducive to the further infiltration of water into the material matrix, resulting in the fracture of hydrolyzable bonds and thus improving the degradation rate [33]. The variation in the water absorption properties of CA film was similar to that of the tow. When the film was degraded in NaOH, the water absorption value was the highest, followed by HCl (increased slowly within 16 weeks), river water, seawater and homemade seawater (no obvious change was found within 16 weeks). During the degradation of CA, the removal of acetyl groups and exposure of hydrogen bonds increased the hydrophilicity of CA and the favorable diffusion into the matrix, thus improving the porosity of cellulose and the accessibility of enzymes, which was conducive to the effective hydrolysis of CA [34,35]. Similar trends were also reported by Wang et al., who studied the degradation of polyglycerol maleate (PGM) films in different environments and found that the increase in water absorption value promoted the diffusion of water molecules through the polymer network and the hydrolysis of ester bonds [36].

### 3.4. FTIR Properties of CA Degraded by Different Aqueous Conditions

**Figure 3:**
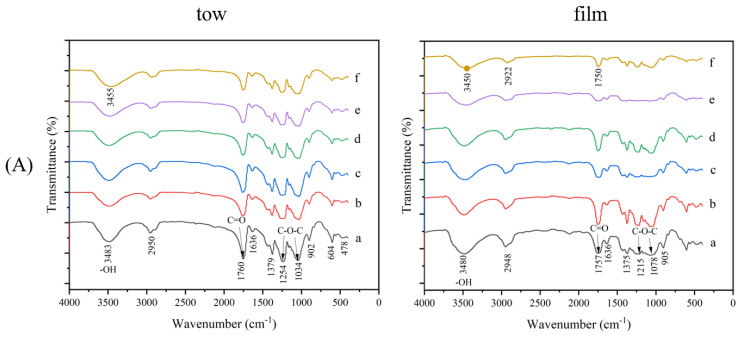

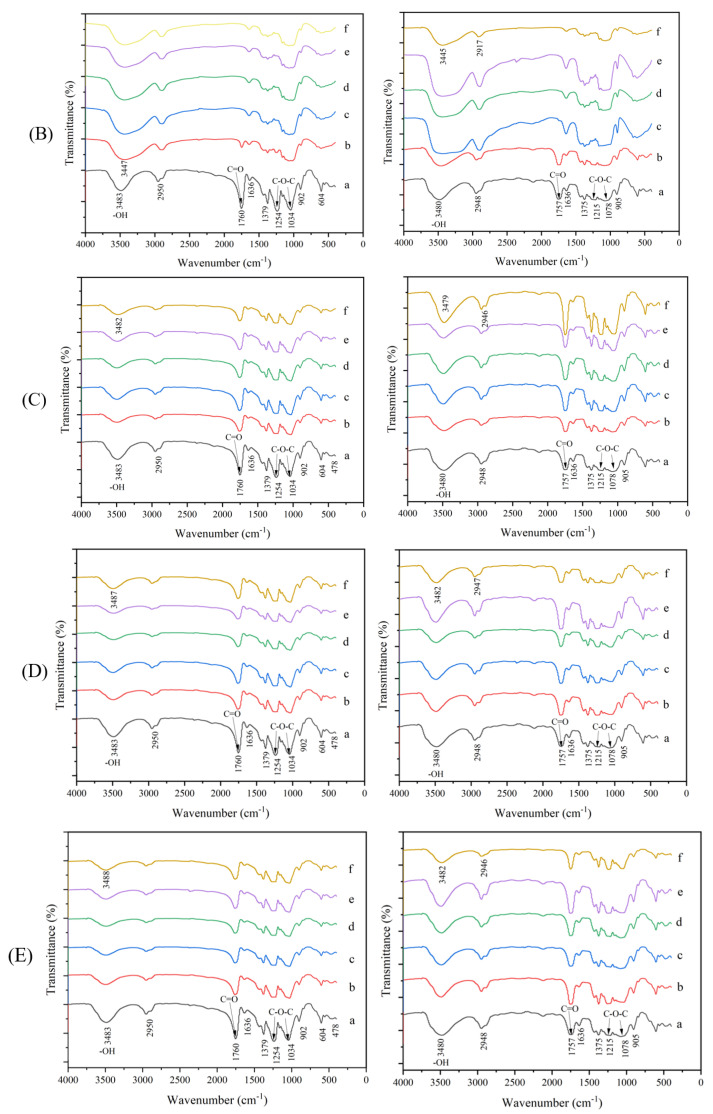
Fourier-transform infrared spectroscopy (FTIR) properties of CA tow and film in different conditions ((**A**) HCl solution; (**B**) NaOH solution; (**C**) river water; (**D**) seawater; (**E**) homemade seawater. Interior of each figure: a: original CA; b, c, d, e, f: degradation for 1, 4, 8, 12, 16 weeks, respectively).

The infrared spectra of tow and film in different degradation conditions are presented in Figure 3. The spectra adsorption peak at 3483 cm^−1^ and 2950 cm^−1^ were attributed to the O–H stretching of hydroxyl groups and C-H stretching vibration of methylene [37,38], respectively. The characteristic absorption peak at 1760 cm^−1^ was derived from C=O stretching vibration [39]. The absorption band at 1379 cm^−1^ was contributed by the C-H stretching vibration of the methyl group, corresponding to the C-H bond in the acetyl group structure [25]. Absorption at 1254 cm^−1^ and 1034 cm^−1^ was assigned to the annular vibration of C-O-C and the skeleton vibration of the C-O-C pyranose ring [40]. The absorption peak at 902 cm^−1^ was produced by C-1 fundamental and ring frequency vibrations, indicating that the cellulose units were connected by β-glucoside bonds [41].

When CA was degraded in the HCl environment, the characteristic absorption peak at 1760 cm^−1^ (C=O) of CA weakened with the increase in degradation time, which could be due to the partial removal of acetyl groups. When CA was degraded in the environment of NaOH, the characteristic absorption peak at 1760 cm^−1^ (C=O) of CA would gradually disappear with the increase in degradation time, while the stretching vibration peak of hydroxyl groups gradually increased, which could be due to the complete removal of acetyl group and the gradual exposure of hydroxyl group in cellulose [42]. However, during the degradation process of CA in river water, seawater and homemade seawater, there was no significant increase or disappearance of absorption peaks, indicating that the molecular structure of CA rarely changed, which was also consistent with the results in Figure 1 and Figure 2.

**Figure 4:**
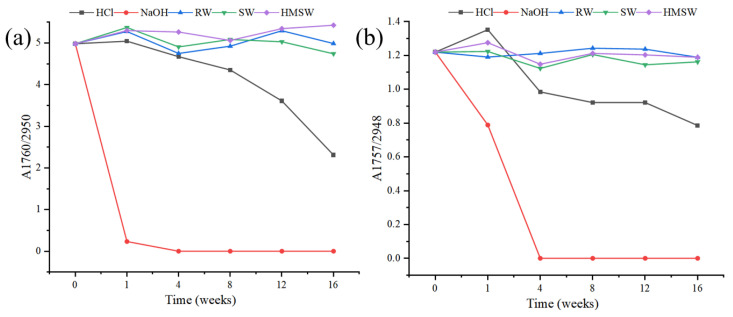
Carbon group change curves of CA filament tow (**a**) and film (**b**) before and after degradation in different conditions.

The relative change in the carbonyl group with degradation time could be expressed by the ratio of the absorption peak intensity of 1760 cm^−1^ to 2950 cm^−1^ (A1760/2950), and the results are shown in Figure 4. It could also be found that the NaOH condition facilitated the strongest degradation effect on both CA tow and film, followed by hydrochloric acid, river water and seawater; homemade sea water was weak.

### 3.5. Morphological Analysis of CA Degraded by Different Aqueous Conditions

**Figure 5:**
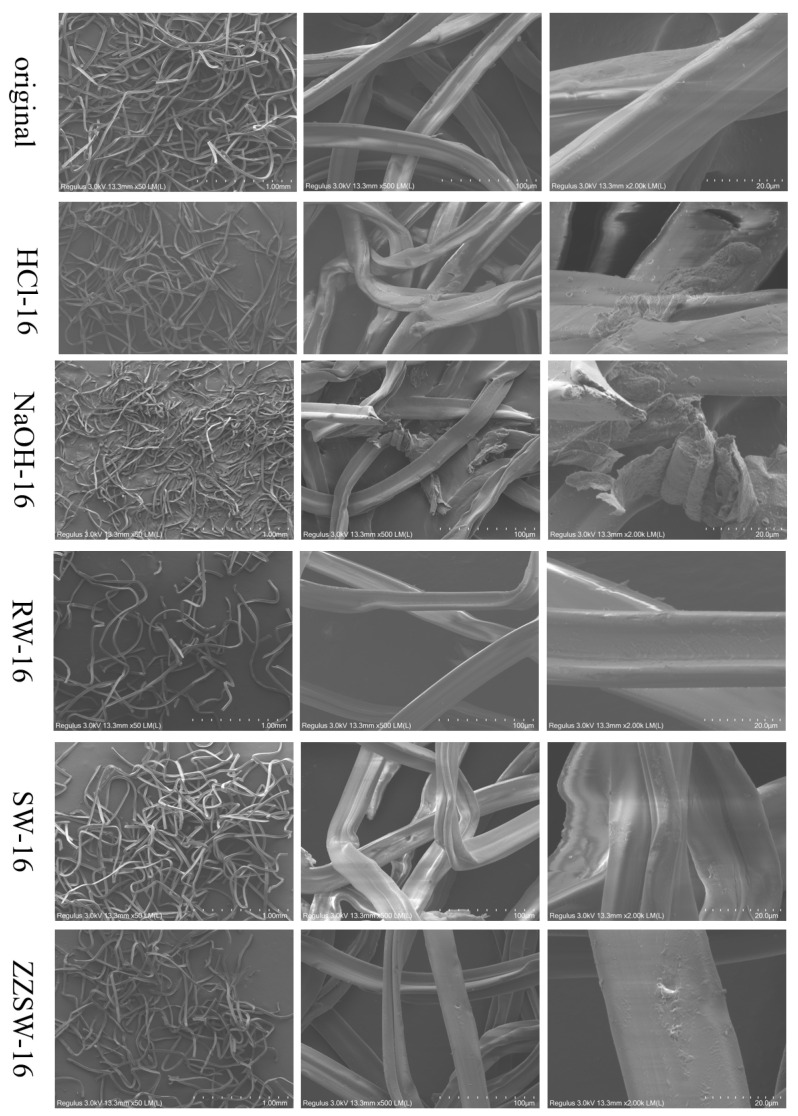
Scanning electron microscopy (SEM) of CA tow degraded in different conditions.

The surface structure of CA tow degraded in different conditions is shown in Figure 5. The surface of the original CA tow was smooth, without deposits or cracks. When CA was exposed to HCl environment, small deposits and micropores were found on the surface of the tow, and the surface of the tow was rough and uneven. When CA was degraded in NaOH environment, a large number of sediments, pores and small convex spots were found on the tow surface, which may be attributed to the fact that alkali treatment would generate more cracks and pores to appear on the surface [43,44]. When CA was degraded in river water, seawater and homemade seawater, the surface of the tow did not change significantly.

**Figure 6:**
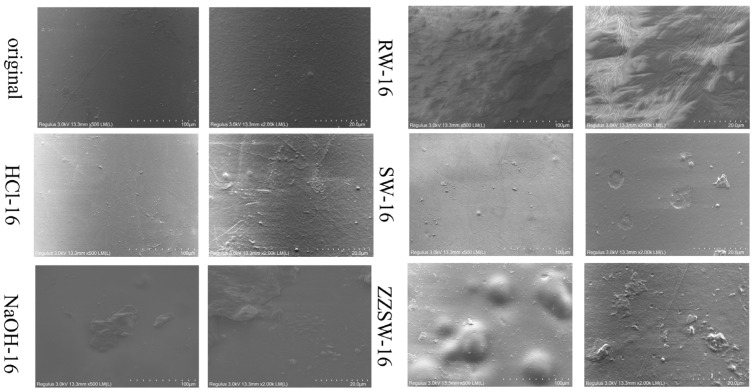
Scanning electron microscopy (SEM) of CA film degraded in different conditions.

The SEM images of CA film degraded in different conditions are shown in Figure 6. The original CA film had a smooth surface morphology without cracks. After HCl treatment, the surface was rough with some ripple marks and uneven surface, which may be caused by the interaction between the HCl solution and the surface of the film. When CA was degraded in the NaOH environment, some deposits were generated on the surface of the film, which may be formed by deacetylation and regeneration of CA film [17].

After the CA film was degraded in the river water, severe surface disruption could be noticed on the film along with a large number of folds and bulges, which might be due to the large number of microorganisms in river water and enzymes secreted by microorganisms constantly attack the film, forming a layer of biofilm on the surface of the film. However, when the CA film was degraded in seawater, a flat and smooth surface could be observed. This result was similar to the previous study by Liu et al., who degraded commercial PET splines in natural seawater for 420 days and found that the morphology of samples did not change significantly, and only a few traces of microbial degradation were observed on the surface [45]. Compared with seawater, the surface of the film was protruding with a small amount of sediment after degradation in homemade seawater, which might be due to the higher content of inorganic salts in homemade seawater than in seawater [46]. A similar result could also be found in the study of Chen et al., who degraded PP1 films in homemade seawater and found that the salt in homemade seawater caused the surface of film to be uneven and cracked [47].

## 4. Conclusions

In summary, the degradation process and properties of CA tow and film in different aqueous conditions were studied. Both CA tow and film could be deacetylated rapidly in NaOH solution within 1 week, and the weight loss rate remained stable afterwards. During degradation in HCl solution, the weight loss rate of both the CA tow and film gradually increased. For river water, seawater and homemade seawater conditions, the degradation of CA tow and film processed very slowly and among which the degradation rate of river water was slightly faster than that of seawater and homemade seawater, which could be due to the higher quantity of microorganisms in river water. With the increase in degradation time, microorganisms and corresponding secreted enzymes would constantly attack the CA matrix and the sample surface would be seriously damaged. Moreover, under the same aqueous condition, the degradation of CA tow was faster than that of film, which might be due to the fact that the contact area between the tow and the aqueous condition was larger, leading to the faster hydrolysis rate. From the above results, it could be seen that the environmental acid-basicity and the presence of microorganisms would greatly accelerate the degradation of CA in the natural environment. Based on the research results presented in this paper, we provided a model for studying the degradation performance and degradation mode of CA in different aqueous conditions and also established a theoretical basis for developing accurate and reliable methods to evaluate the degradation performance of CA.

## Figures and Tables

**Table 1:** Chemical composition of homemade seawater [19].

Composition	NaCl	MgCl_2_	Na_2_SO_4_	CaCl_2_	KCl	NaHCO_3_	KBr
Concentration (mg/mL)	24.53	5.2	4.1	1.16	0.695	0.201	0.101

Note: pH is 8.2.

**Table 2:** Degree of substitution of CA tow and film in different conditions.

Time	HCl Solution (pH = 1)		NaOH Solution (pH = 12)		River Water		Seawater		Homemade Seawater	
	Tow	Film	Tow	Film	Tow	Film	Tow	Film	Tow	Film
1 week	2.34 ± 0.02 ^a^	2.37 ± 0.07 ^a^	0.27 ± 0.06 ^a^	1.72 ± 0.06 ^a^	2.42 ± 0.03 ^a^	2.40 ± 0.04 ^a^	2.39 ± 0.01 ^a^	2.41 ± 0.03 ^abc^	2.39 ± 0.01 ^a^	2.40 ± 0.03 ^a^
3 weeks	2.28 ± 0.01 ^a^	2.38 ± 0.00 ^a^	0.06 ± 0.01 ^b^	0.01 ± 0.01 ^b^	2.35 ± 0.04 ^ab^	2.37 ± 0.04 ^a^	2.37 ± 0.06 ^ab^	2.39 ± 0.03 ^abc^	2.38 ± 0.01 ^ab^	2.38 ± 0.02 ^ab^
4 weeks	2.27 ± 0.02 ^ab^	2.37 ± 0.02 ^a^	0	0	2.35 ± 0.00 ^ab^	2.40 ± 0.03 ^a^	2.38 ± 0.01 ^a^	2.40 ± 0.02 ^ab^	2.35 ± 0.02 ^abc^	2.38 ± 0.03 ^ab^
5 weeks	2.18 ± 0.02 ^bc^	2.36 ± 0.00 ^a^	0	0	2.37 ± 0.04 ^ab^	2.41 ± 0.03 ^a^	2.40 ± 0.04 ^a^	2.39 ± 0.05 ^abc^	2.35 ± 0.04 ^abc^	2.38 ± 0.01 ^ab^
7 weeks	2.13 ± 0.02 ^cd^	2.34 ± 0.02 ^a^	0	0	2.35 ± 0.00 ^ab^	2.39 ± 0.06 ^a^	2.38 ± 0.03 ^ab^	2.42 ± 0.00 ^a^	2.34 ± 0.01 ^abc^	2.35 ± 0.03 ^ab^
8 weeks	2.08 ± 0.07 ^d^	2.33 ± 0.02 ^a^	0	0	2.35 ± 0.02 ^ab^	2.37 ± 0.04 ^a^	2.39 ± 0.04 ^a^	2.40 ± 0.02 ^abc^	2.35 ± 0.02 ^abc^	2.35 ± 0.02 ^ab^
9 weeks	2.04 ± 0.08 ^d^	2.24 ± 0.01 ^b^	0	0	2.35 ± 0.03 ^ab^	2.37 ± 0.02 ^a^	2.38 ± 0.06 ^ab^	2.39 ± 0.03 ^abc^	2.35 ± 0.05 ^abc^	2.36 ± 0.00 ^ab^
11 weeks	1.89 ± 0.03 ^e^	2.15 ± 0.03 ^bc^	0	0	2.36 ± 0.04 ^ab^	2.36 ± 0.03 ^a^	2.37 ± 0.04 ^ab^	2.35 ± 0.05 ^abc^	2.31 ± 0.03 ^abc^	2.34 ± 0.03 ^ab^
12 weeks	1.84 ± 0.01 ^ef^	2.10 ± 0.02 ^c^	0	0	2.33 ± 0.02 ^ab^	2.32 ± 0.03 ^ab^	2.37 ± 0.03 ^ab^	2.37 ± 0.02 ^abc^	2.32 ± 0.02 ^abc^	2.34 ± 0.02 ^ab^
14 weeks	1.76 ± 0.07 ^f^	1.85 ± 0.08 ^d^	0	0	2.29 ± 0.01 ^b^	2.32 ± 0.01 ^ab^	2.37 ± 0.04 ^ab^	2.34 ± 0.02 ^bc^	2.29 ± 0.03 ^bc^	2.35 ± 0.02 ^ab^
16 weeks	1.33 ± 0.01 ^g^	1.78 ± 0.06 ^d^	0	0	2.16 ± 0.04 ^c^	2.24 ± 0.02 ^b^	2.30 ± 0.01 ^b^	2.32 ± 0.03 ^c^	2.28 ± 0.03 ^c^	2.32 ± 0.01 ^b^

Experimental data are expressed as mean ± standard deviation (mean ± SD, n = 3). Different letters (a–g) in the same column represent significant differences (*p* < 0.05).

## Data Availability

The data presented in this study are available on request from the corresponding author.
